# Solid-State Luminescence of an Americium(III) β‑Diketonate Complex: Structure and Spectroscopic Characterization of [Am(acac)_2_(μ-acac)(H_2_O)]_2_

**DOI:** 10.1021/acs.inorgchem.6c01922

**Published:** 2026-08-23

**Authors:** Thomas L. McCusker, David Schnable, Ana Arteaga, Jeffery A. Bertke, Robert G. Surbella, Karah E. Knope

**Affiliations:** † Department of Chemistry, 8368 Georgetown University, 37th and O Streets NW, Washington, District of Columbia 20057, United States; ‡ Pacific Northwest National Laboratory, 902 Battelle Boulevard, Ri chla nd, Washington 99354, United States

## Abstract

Luminescence spectroscopy is a powerful method for probing the chemical and physical properties of metal ions, as perturbation of electronic states due to ligand–field effects yields changes in emissive behavior. This sensitivity is particularly valuable for the trivalent actinides, whose chemical bonding is still not well understood. Here, we report the synthesis and characterization of an americium β-diketonate, [Am­(acac)_2_(μ-acac)­(H_2_O)]_2_ (**Am**_**2**_); acac = acetylacetonate. **Am**_**2**_ was prepared via slow evaporation of a methanolic americium–acac solution and characterized via single-crystal X-ray diffraction, Raman, UV–vis–NIR absorbance, and luminescence spectroscopies. The structure consists of acac-bridged Am dimers with both Am­(III) metal centers adopting a distorted square antiprismatic coordination geometry. **Am**_**2**_ represents a rare example of ligand-sensitized Am­(III) emission in the solid state, with ligand excitation at 320 nm resulting in characteristic 5f–5f emission bands at ca. 699, 850, and 1060 nm that correspond to the ^5^D_1_^′^ → ^7^F_1–3_^′^ transitions. The absence of the ^5^D_1_^′^ → ^7^F_0_^′^ transition is consistent with the *C*_1_ symmetry about the metal center established from structural studies. The observed peak splitting and bathochromic shifting can be attributed to crystal-field effects and a limited degree of covalency in Am–acac bonding.

## Graphical abstract

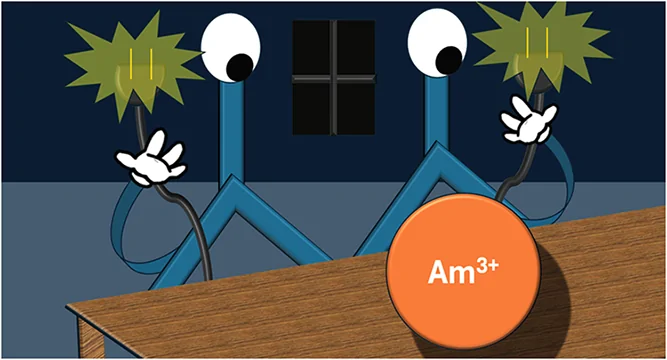

## Introduction

Nuclear energy and national security have driven significant research into the chemical and physical properties of the actinides over the last 50 years.^1^ Much of this attention has focused on the early actinides, including thorium, uranium, and to a lesser extent neptunium and plutonium, due to their direct application in energy production, spent fuel management, and nuclear security as well as the inventories and scale at which these elements can be handled.^2,3^ More recently, expansion of radiological facilities and advances in characterization methods have enabled the synthesis and interrogation of structure–property relationships for the later trivalent actinides, including curium and americium.^4^ As such, the speciation, bonding, and overall chemical behavior of these elements has gained increased attention.^4–13^ These efforts are driven not merely by synthetic accessibility, but the long-recognized importance of managing nuclear fuel cycle byproducts and understanding their chemical and physical behavior. For example, the bulk of residual radioactivity in ca. 100-year-old spent nuclear fuel is projected to stem from long-lived americium isotopes (^241^Am, ^242m^Am, and ^243^Am).^14^

The early (Th–Pu) and later actinides (Am–Lr) have distinct chemical behavior. The early actinides exhibit rich redox properties unique to each element, with metal ion oxidation state largely governing coordination behavior, charge density, and overall speciation.^3^ Yet, across the actinide series, poor intrashell electron shielding produces a steady contraction of the 5f orbitals such that the electrons of the later actinides are widely considered localized compared to the more itinerant electrons of their early actinide counterparts.^15^ Ultimately, this has led to the perception that the bonding interactions of the later actinides are primarily electrostatic in nature, closely resembling that of the lanthanides.^16–18^ This chemical similarity between the trivalent lanthanides and actinides, arising from comparable ionic radii and coordination chemistry, has motivated the use of lanthanides as non-radioactive surrogates for the trivalent actinides, yet it also makes their separation from irradiated nuclear fuels a major bottleneck for spent fuel management and storage.^19^ Understanding the nuances that underpin differences in the chemical behavior of the trivalent lanthanides and actinides is therefore critical to controlling their chemistry in nuclear fuel, separations, and environmental remediation contexts.^19,20^

Several analytical strategies have been employed to probe actinide electronic structure and bonding including X-ray absorption spectroscopy (XAS), high-energy-resolution X-ray absorption near-edge spectroscopy (HR-XANES), and resonant inelastic X-ray scattering (RIXS).^13,21–23^ Importantly, these experimental methods are often coupled with theoretical investigations.^16,24–26^ As an example, Cross et al. examined bonding in europium and americium chloride-based complexes and found slight differences in the mixing of the chloride 3p orbitals with the Eu­(III) 4f orbitals versus the Am­(III) 5f orbitals.^27^ Although negligible mixing of the 3p and 4f-orbital contribution was observed for Eu­(III), the 5f-orbitals of Am­(III) displayed 0.54(5)% 3p character, likely due to more pronounced radial extension of the 5f orbitals. This is admittedly a slight difference; however, previous reports have demonstrated that effective separation of Eu­(III) and Am­(III) rely on just single kcal differences in binding affinities.^28^ As a result, in addition to X-ray and computational methods, optical spectroscopies, that are sensitive to changes in electronic structure, have attracted increased focus particularly for the actinides.^7,9,10,29–34^ Am­(III) luminescence arises from intrashell 5f–5f transitions that are sensitive to local symmetry and ligand field effects.^31,33^ Consequently, photoluminescence spectroscopy can be leveraged to probe the extent of metal–ligand orbital overlap or mixing in various Am­(III) systems.^9,35^

Photoluminescence requires an accessible emissive excited state. Am­(III) exhibits absorption features almost exclusively stemming from intraconfigurational 5f–5f transitions that are typically characterized by low molar absorptivities compared to Laporte-allowed transitions.^36,37^ This weak intrinsic absorption intensity limits the efficacy of direct-metal excitation. Yet in lanthanide systems, this limitation has been circumvented via antenna sensitization, wherein a strongly absorbing ligand harvests excitation energy and transfers it to the metal center.^9,38^ Using this approach, efficient energy transfer can be achieved provided the triplet state energy level of the ligand is suitably positioned relative to receiving energy levels of the metal center. Despite broad success of this strategy for the lanthanides, relatively few examples of antenna sensitization of americium have been reported.^9,33^ With this in mind, we sought to leverage the antenna effect to probe the electronic structure of Am­(III). Members of the β-diketonate family were selected given their well-established sensitization of lanthanide ions, well-characterized triplet states, and ease of functionalization.^39,40^ This work focuses on the simplest member, acetylacetonate (acac). Reaction of Am­(III) with acac in methanol readily yielded **Am**_**2**_, [Am­(acac)_2_(μ-acac)­(H_2_O)]_2_. This phase displayed remarkable luminescent properties, including several emissive transitions seldom observed in the solid state.^30^ Taken together, structural analysis and characterization of the luminescence behavior of **Am**_**2**_, including the absence of the ^5^D_1_^′^ → ^7^F_0_^′^ emissive band, the bathochromic shifting of the ^5^D_1_^′^ → ^7^F_1_^′^ band, and the unusually well-resolved band splitting, provides insights into the effect of metal ion coordination geometry, electronic structure, and bonding on Am­(III) emission.

## Results and Discussion

### Synthesis and Structure Description of **Am_2_**

Compound **Am**_**2**_, [Am­(acac)_2_(μ-acac)­(H_2_O)]_2_, was prepared by dissolving AmCl_3_·*n*H_2_O in methanol, followed by the addition of acetylacetone and triethylamine in a 1:5:7.5 (metal/ligand/base) molar ratio. Evaporation of the solvent in a radiological glovebox yielded orange crystals of **Am**_**2**_ within 12 hours (Figure S1).

The structure of **Am**_**2**_ was determined using single-crystal X-ray diffraction (scXRD) and was found to be isomorphous with a Nd­(III)–acac compound previously reported.^41^ The compound crystallizes in the triclinic space group *P*1̅, and the structure consists of a dimeric unit, where two Am­(III) metal centers are bridged by two acac ligands (Figures 1 and S2). The asymmetric unit of the structure consists of one-half of the dimer. The Am---Am distance in the dimer is 4.132(10) Å. Overall, the metal centers are 8-coordinate and adopt a distorted square antiprismatic coordination geometry with *C*_1_ local symmetry. The twist angle of the polyhedron, defined here as the degree to which the top and bottom plane of the antiprism are offset, is 44.8°. In addition to binding three oxygen atoms from the two bridging μ-acac ligands, each Am­(III) metal center is also coordinated to five oxygen atoms from two κ^2^-acetylacetonate ligands and one water molecule. Average Am–O bond distances for bridging–O, κ_bridged_^2^–O, and κ_nonbridged_^2^–O are 2.539(5), 2.410(5), and 2.405(5) Å, respectively. The Am–O_water_ distance is 2.455(5) Å. The Nd­(III) analogue exhibits similar bond lengths to the Am­(III) complex, and the same twist angle. Average Nd–O_acac_ bond lengths are 2.538(4), 2.416(4), and 2.405(5) Å for bridging–O, κ_bridged_^2^–O, and κ_nonbridged_^2^–O, respectively. The Nd–O_water_ bond length is 2.460(2) Å.^41^ The powder X-ray diffraction pattern collected for the reaction product of **Am**_**2**_ showed good agreement with that calculated from scXRD data, indicating that the phase is representative of the bulk product (Figure S3). However, it is worth noting that **Am**_**2**_ co-crystallized with a triethylamine hydrochloride (TEA·HCl) salt.

**Figure 1:**
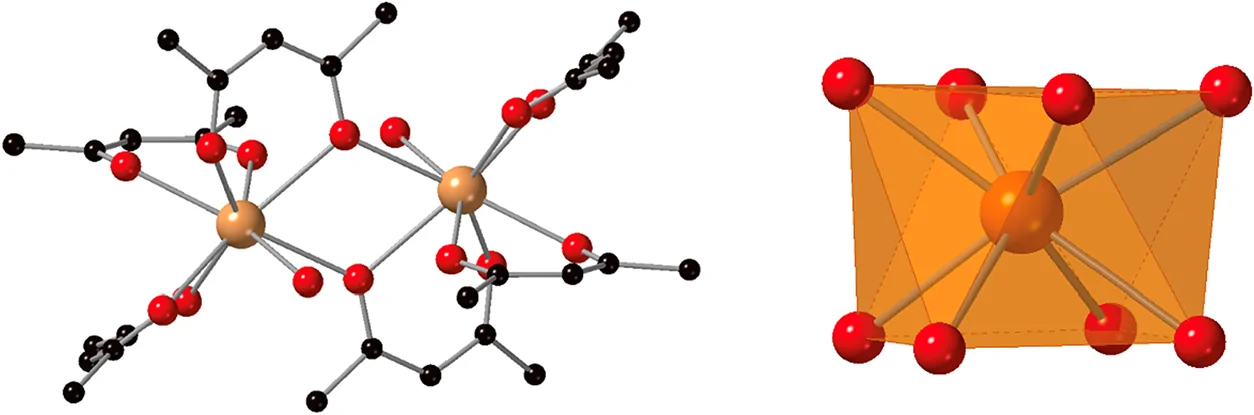
Left: Ball and stick illustration of the dimeric complex, [Am­(acac)_2_(μ-acac)­(H_2_O)]_2_. Right: Polyhedral representation of the 8-coordinate Am­(III) metal centers. The Am atoms are shown in orange, oxygen in red, and carbon in black. Hydrogen atoms have been omitted for clarity.

## Raman Spectroscopy

The Raman spectrum of **Am**_**2**_ (Figure 2) was collected to examine how similarities in bonding (i.e., bond metrics established from single-crystal X-ray diffraction) between **Am**_**2**_ and the Nd­(III) analogue manifest in the vibrational properties of the compounds.^41^ The 120–2000 cm^–1^ region of the spectrum was deconvoluted using best-fit Voigt functions to determine peak positions (Figure S5). Several bands in the Raman vibrational spectrum, e.g., at 537.0, 569.2, 655.7, and 1021.5 cm^–1^, are attributed to the acetylacetonate ligands.^42,43^ For the Nd­(III) analogue, a peak centered at 406 cm^–1^ has previously been assigned as a Nd–O_acac_ vibration.^42^ Notably, the spectrum of **Am**_**2**_ exhibits a band in the same region at 411.4 cm^–1^ that similarly can be assigned to a Am–O_acac_ vibration. The similarities for bands attributed to the M–O_acac_ vibrational modes in the Raman spectra of the Am­(III) and Nd­(III) are consistent with the similarities in bond distances from Am­(III) to Nd­(III). However, it should be mentioned that these bands overlap with C–CH_3_ vibrational modes reported for acetylacetone^44^ and, as such, may not be diagnostic for differences in bonding.

**Figure 2:**
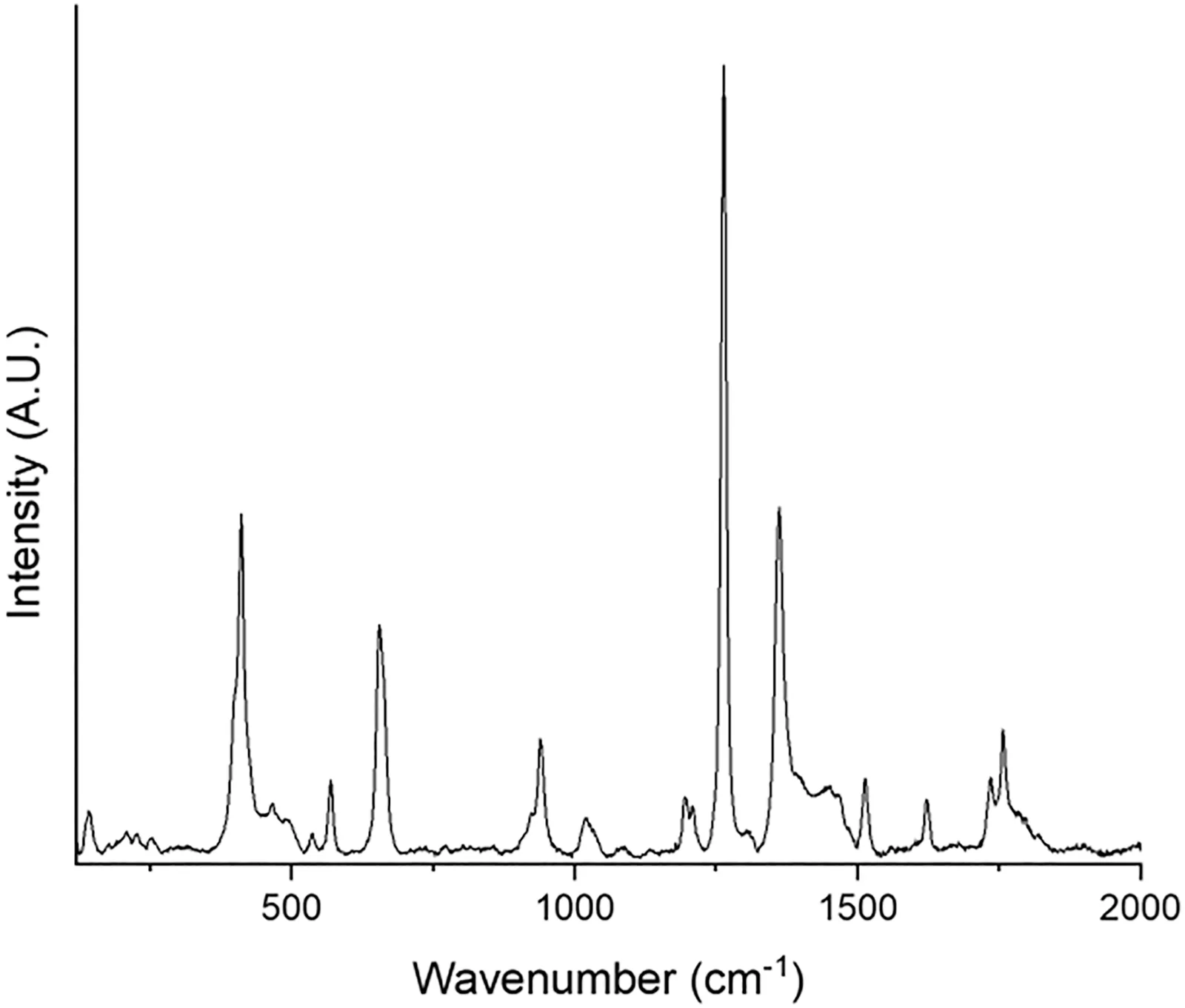
Raman spectrum of **Am**_**2**_ collected at room temperature using a 532 nm incident laser.

### UV–Vis–NIR Absorption Spectroscopy

Samples of microcrystalline **Am**_**2**_ were sufficiently transpicuous to enable direct collection of transmittance spectra in the 300–1200 nm region (Figure 3). A broad transition is present between 300 and 400 nm, near the edge of the collection range, which arises from the ligand π → π* transition.^45,46^ Multiple sharp absorption bands were also observed in the UV–visible–NIR regions that are attributed to the intraconfigurational 5f–5f transitions of Am­(III).^35,37,47,48^ In the UV region, the absorption data were sufficiently well resolved to enable deconvolution of each absorption band up to the ^5^D_3_^′^ **←** ^7^F_0_^′^ transition at ca. 400 nm (Figures S6–S9). Bands in this region are attributed to transitions from the ^7^F_0_^′^ ground state to any of multiple closely spaced excited states (*e*.*g*., ^5^H_3–5_^′^, ^5^D_2_^′^, ^5^G_3_^′^). Tentative transition assignments are provided in Figure 3. The prominent ^5^L_6_^′^ ← ^7^F_0_^′^ transition, which has been reported to occur between 500 and 520 nm, is present at ca. 504 nm, followed by three NIR transitions at ca. 830, 940, and 1030 nm which are assigned as F6,5,4′7←F0′7 , respectively.^8,35,49–51^

**Figure 3:**
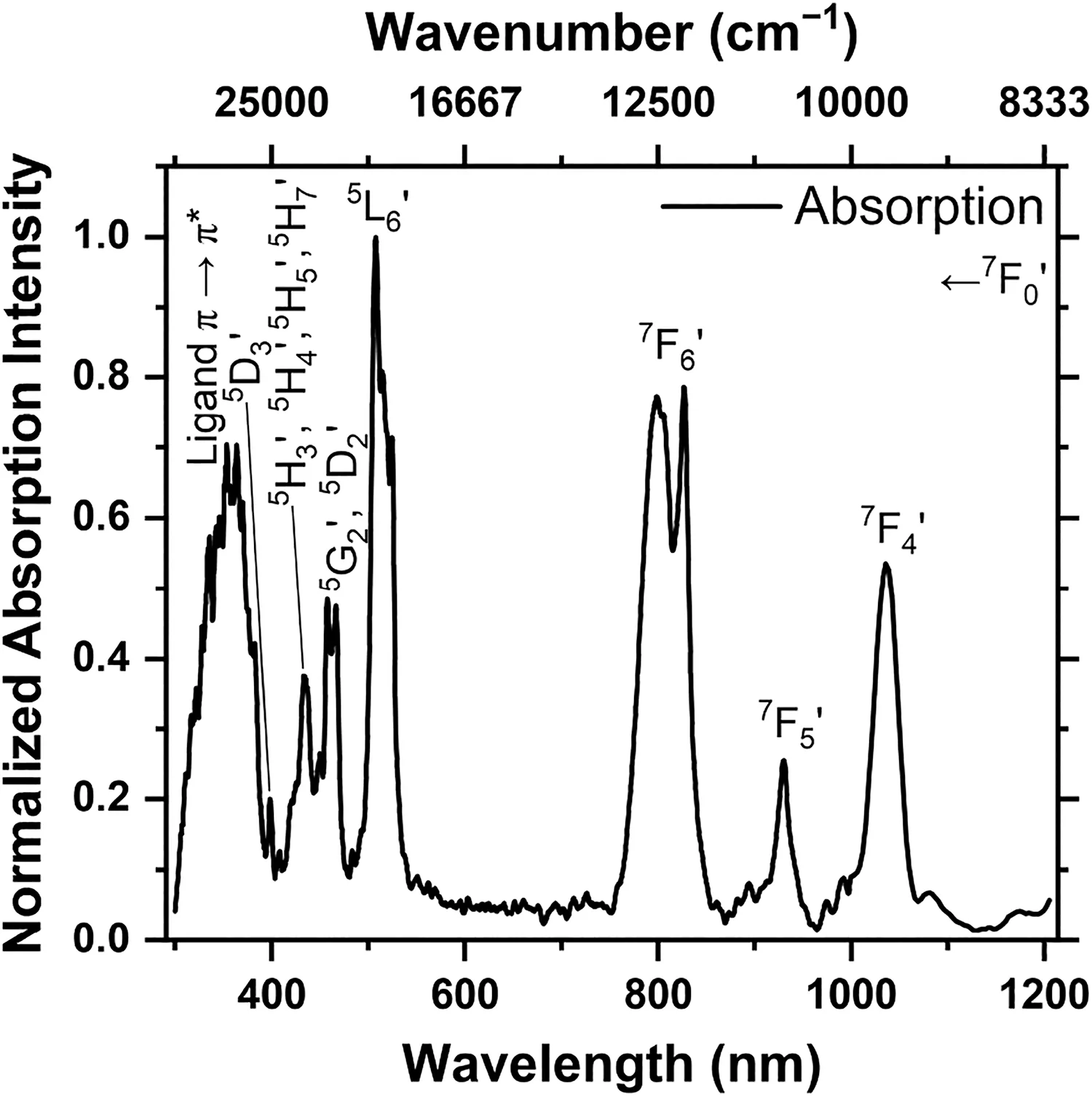
Solid-state UV–vis–NIR absorption spectrum of **Am**_**2**_.

### Photoluminescence Spectroscopy

There are a limited number of reports of Am­(III) emission from single crystals.^9,30,31,33^ Similar to the trivalent lanthanide ions, the 5f–5f transitions of Am­(III) are Laporte-forbidden, resulting in relatively weak absorption bands that limit direct-metal excitation.^36^ To circumvent weak absorption, organic ligands (i.e., acac) can be leveraged to sensitize Am­(III) emission. For **Am**_**2**_, excitation within the acac absorption envelope at 320 nm, results in characteristic 5f–5f emission bands at ca. 699, 850, and 1060 nm that correspond to the ^5^D_1_^′^ → ^7^F_1–3_^′^ transitions, respectively (Figure 4).

**Figure 4:**
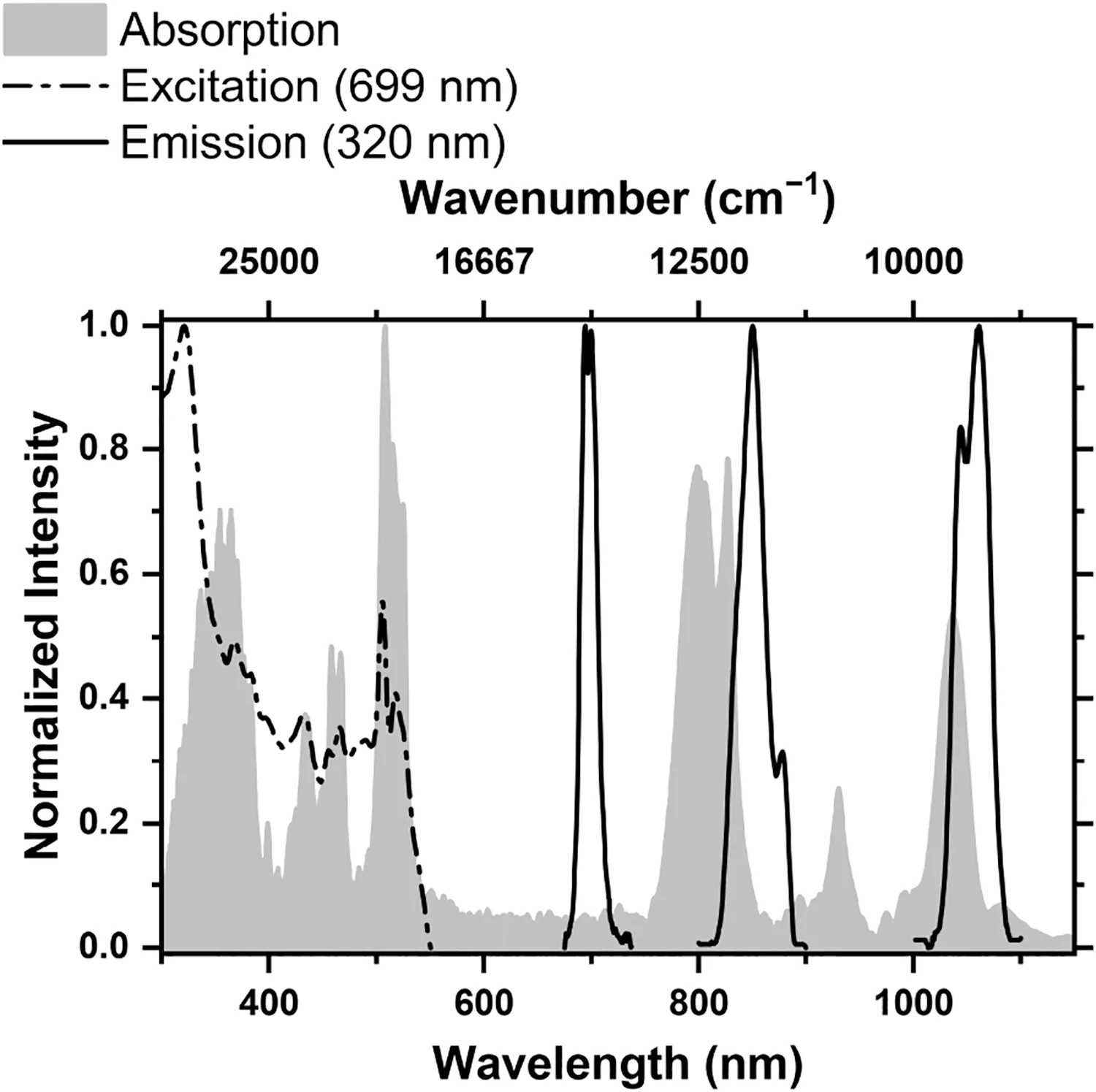
UV–vis–NIR absorption spectrum (shown in gray) overlaid with the excitation (dashed black trace) and emission (solid black trace) spectra of **Am**_**2**_ single crystals.

The energy gap between the triplet state of the supporting acac ligand (25,300 cm^–1^) and the ^5^D_1_^′^ emissive state of the Am­(III) metal center (19,230 cm^–1^) is slightly outside the range established for efficient energy transfer based on Latva’s empirical rule (Δ*E* = 6080 cm^–1^).^52^ As such, higher energy levels, such as the ^5^D_2_ or ^5^H_5_ levels, could be involved in the energy transfer process. The primary ^5^D_1_ emissive level could presumably thereafter be populated through nonradiative decay. The first transition observed from this emissive energy level, the D1′5→F1′7 transition, has been noted to exhibit ligand field-induced shifting, occurring anywhere from 670 to 720 nm (14,925–13,889 cm^–1^).^10,30,32,35,51^ The ^5^D_1_^′^ → ^7^F_1_^′^ transition of **Am**_**2**_ is located at 699 nm and displays a relatively small bathochromic shift that is consistent with the nephelauxetic effect and is indicative of slight orbital mixing compared to the free ion.^48,53,54^

All three of the observed emission bands in **Am**_**2**_ are multicomponent, featuring multiple sub-transitions likely due to Stark**-**type crystal-field splitting. Stark splitting refers to the breakdown of *j* degeneracy as a result of the ligand-induced electric field surrounding the metal ion. Each *j* state splits into a maximum of 2*j* + 1 *m*_*j*_ sublevels, referred to as Stark levels, depending on the symmetry of the surrounding ligand field. Electronic transitions are possible from each Stark level within the emissive manifold to each of those in the receiving manifold, *e*.*g*., the ^5^D_1_^′^ → ^7^F_1_^′^ transition (*j* = 1, *j*′ = 1) is expected to split into nine Stark levels in this low symmetry coordination environment; transitions from ^5^D_1_^′^ to ^7^F_2_^′^ or ^7^F_3_^′^ will contain even more components.^55^ Due to vibrational broadening, it is uncommon for Stark levels to be well resolved, especially at room temperature.

The Stark splitting observed from **Am**_**2**_ is unusually well resolved for Am­(III) at room temperature; this is plausibly a product of a strong ligand field, rigid solid-state coordination environment, and efficient ligand-sensitized emission (enabling narrow experimental bandpasses). The fine resolution observed in these spectra allowed deconvolution of each emission band into 4–6 sub-components (Figures S10–S12). The ^5^D_1_^′^ → ^7^F_1_^′^ transition was modeled as six distinct components centered at 685, 689, 693, 698, 703, and 707 nm. The ^5^D_1_^′^ → ^7^F_2_^′^ and D1′5→F3′7 transitions consisted of four components, centered at 833, 850, 866, and 879 nm for the former and 1041, 1043, 1060, and 1070 nm for the latter.

Notably absent from the emission spectrum of **Am**_**2**_ is the nominally 600 nm emission band corresponding to the D1′5→F0′7 transition. For the limited examples of solid-state Am­(III) luminescence available for comparison, it is often the case that this emission band is absent.^10,32,34^ Previous studies have correlated these systematic absences to the symmetry of the Am­(III) metal center. Specifically, 8-coordinate Am­(III) metal centers that exhibit *C*_2*v*_ symmetry as well as 7, 8, and 9-coordinate metal centers with *C*_1_ site symmetry often lack a ^5^D_1_^′^ → ^7^F_0_^′^ emission band (or it is weak).^30^ Suppression of the D1′5→F0′7 transition in **Am**_**2**_ is consistent with these previous reports, as the overall symmetry of the Am­(III) metal center is approximately *C*_1_ owing to a slight distortion from a perfect square antiprism geometry and the inequivalence of the oxygen atoms.

## Conclusion

In summary, a novel americium dimer, [Am­(acac)_2_(μ-acac)­(H_2_O)]_2_, supported by acetylacetonate is reported. The compound is isomorphous with a previously reported lanthanide–acac phase and was isolated under relatively similar synthetic conditions.^41^ Examination of the luminescent properties of **Am**_**2**_ provides insights into the electronic structure of Am­(III). The compound exhibits ligand-sensitized metal centered emission enabled by acetylacetonate, with three Am­(III) emission bands resulting from intraconfigurational 5f–5f transitions observed. All three of these emissive transitions have multicomponent features arising from crystal-field splitting that are sufficiently resolved to enable peak deconvolution. Finally, a limited degree of orbital mixing between the americium and acetylacetone ligands is evidenced by a slight bathochromic shift of the emissive transitions compared to the free ion. Ultimately, this compound is a key example of solid**-**state americium luminescence and expands on our limited knowledge of the electronic structure of this transuranic element.

## Experimental Methods

### Caution

^243^Am is a hazardous radiological isotope (*t*_1/2_ = 7370 years) and a potent α and γ emitter (0.2 Ci/g, α decay energy = 5.438 MeV). Furthermore, ^239^Np is a daughter product of ^243^Am and is a potent β emitter (2.3 × 10^5^ Ci/g, β decay energy = 0.722 MeV). Prior to the synthesis, the americium stock was processed via ion-exchange chromatography to remove daughter products. The material should be handled only by trained personnel in a radiological facility equipped for handling hazardous transuranic isotopes. Extreme care must be taken to ensure that radiological handling procedures, engineered controls, and containment/packaging guidelines are sufficient for safe conduct of work.

#### Synthesis of **Am**_**2**_, [Am­(acac)_2_(μ-acac)­(H_2_O)]_2_

An Am­(III) chloride solution was prepared by dissolving 30.8 mg of Am­(NO_3_)_3_·*x*H_2_O in concentrated HCl_(aq)_. The resulting solution was concentrated to near dryness and reconstituted three times to decompose residual nitrate to a noxious gas. The resulting residue was redissolved in 776 μL of concentrated HCl_(aq)_ to produce a ^243^Am stock solution (Figure S1). The concentration of the stock solution was determined to be 39.7 mg/mL using γ-spectroscopy. A 227 μL aliquot of the ^243^Am stock (9.0 mg ^243^Am, 37 μmol) was transferred to a 5 mL conical microdistillation vial and gently boiled to dryness while stirring. The resulting solid residue was dissolved in 1 mL of methanol (MeOH) yielding a pale pink solution (Figure S1), which was then transferred by pipet to a 1**-**dram scintillation vial. Acetylacetone (19.0 μL, 18.5 mg, 185 μmol, 5.0 equiv) was added, followed by triethylamine (38.8 μL, 28.2 mg, 278 μmol, 7.5 equiv). Upon the addition of triethylamine, the solution became turbid and orange in color (Figure S1). The reaction vial was then placed, uncapped, in an ∼10 cm tall secondary vial to limit the rate of solvent evaporation, and left overnight in a negative pressure glovebox. Orange spindle-like crystals of **Am**_**2**_ were observed after concentrating for approximately 12 h.

### Single-Crystal X-ray Diffraction (scXRD)

Crystals of **Am**_**2**_ were removed from the reaction vial and placed on a glass slide in mineral oil. A single crystal was selected based on visual quality and affixed to a MiTeGen micromount; a cap of heat-sealed plastic tubing was then adhered to the mount to provide additional radiological containment.^12^ Single-crystal X-ray diffraction data were collected on a Bruker D8 Venture Diffractometer, equipped with a Mo IμS X-ray source (λ = 0.71073 Å), and a Photon II CCD detector. Data were processed with the APEX III software suite. Data were reduced using SAINT, and absorption corrections were applied using SADABS.^56,57^ The structure was solved via direct methods using intrinsic phasing and refined using ShelXle.^58–60^ Further details of the refinement are provided in the Supporting Information.

### Powder X-ray Diffraction (pXRD)

Powder X-ray diffraction data were collected using a Rigaku Ultima IV diffractometer (Cu Kα, λ = 1.524 Å, 2θ = 3–40°) (Figure S3). Prior to data collection, the sample was placed on a crystalline silicon (“zero-background”) plate within a Bruker PMMA pXRD mount and contained under a Kapton polyimide film. pXRD data were collected ten times over 2θ = 3–40° and then averaged for noise reduction. Simulated diffraction patterns were generated for comparison from single**-**crystal diffraction data.

### Raman Vibrational Spectroscopy

Normal-Stokes Raman scattering spectra were collected on single crystals (Figure S4). Crystals were staged between two quartz windows in a custom three-dimensional (3D)-printed sample holder to provide radiological containment. The Raman spectrum was collected using a Renishaw inVia confocal Raman microscope using a 5× magnification objective. Incident light was provided by a 532 nm class 3B laser (Renishaw RL-532, 500 mW continuous power). Transmitted laser power was reduced using a series of neutral density filters resulting in nominally 12.5% light transmission to the sample. Scattered light was projected across a Renishaw Centrus CCD detector via a motorized variable-angle diffraction grating (1800 grooves/mm). The spectrum required multiple collections and due to wide spectral range were collected in segments and stitched together using Renishaw’s SynchroScan technology. Prior to data collection, wavenumber calibration was referenced to the Raman scattering band of an internal silicon standard (520.5 cm^–1^).^61^

### Absorption Spectroscopy

Transmission measurements were collected from microcrystalline material using a CRAIC 2030 PV microspectrophotometer affixed to a Zeiss Axioscope 5 microscope. Sample containment was identical to that employed for Raman vibrational spectroscopy. All measurements were collected at 50× magnification over the 300–1250 nm region. Transmission measurements from multiple single crystals were collected and averaged for noise reduction. Data were converted to absorption units via the equation Abs = 2 – log­(% *T*) (Figure 3).

### Photoluminescence Spectroscopy

A bulk sample of **Am**_**2**_ was placed in a welled solid-state emission sample holder and was contained using a quartz coverslip and vinyl tape. Emission spectra were collected on a Horiba NanoLog spectrometer, using the FluorEssence software suite, version 3.9.0.1. Data were collected using an S-side FL-1073 detector (covering the 450–900 nm region) or a T-side iHR320 with DSS-IGA-IR-1.7 detector at the lateral exit (covering the 950–1250 nm region). The well plate sample was secured upright in a variable-angle solid sample stage and oriented 35° from the axis normal to the plane of the exit port, (i.e., 125° from the axis normal to the excitation entrance port). The slightly shallower angle (than the idealized 135° for right angle mode) was employed to minimize specular reflection of the excitation light toward the detector. Emission measurements in the 500–900 nm region were recorded using the FL-1073 detector and a diffraction grating emission monochromator with 1200 grooves/mm and 500 nm blaze. Where appropriate, a 550 nm long-pass filter was used in the S-side emission path to preclude observation of second-order diffraction artifacts. Measurements in the NIR region (1000–1100 nm) were recorded using the liquid nitrogen cooled DSS-IGA-IR-1.7 detector and emission monochromator diffraction gratings with 600 grooves/mm and 1000 nm blaze. For the NIR spectrum, a 780 nm long-pass filter was placed in the emission path at the entrance to the iHR320 to preclude observation of second-order diffraction artifacts. For all spectral data collection, monochromatic excitation light was provided by a broad-spectrum xenon arc lamp and diffraction grating monochromator with 1200 grooves/mm and 300 nm blaze. The excitation data were collected while monitoring emission at 699 nm with excitation/emission monochromator bandpasses of 5 and 14.7 nm, respectively. The visible-region (450–750 nm) emission data were collected under excitation at 320 nm with excitation/emission monochromator bandpasses of 7.5 and 2 nm, respectively. The NIR-region (750–1200 nm) emission data were collected under excitation at 320 nm with excitation/emission monochromator bandpasses of 14.7 and 10 nm, respectively (Figure 4). Peak deconvolution of emission peaks was performed in OriginPro software.^62^

## Supplementary Material



## Notes

Photographs of the reaction solutions and **Am_2_** crystals, crystal structure refinement details, thermal ellipsoid plot, powder X-ray diffraction patterns, and additional vibrational and optical spectra (PDF)
