# Supplementary material for: Solid-State Luminescence of an Americium(III) β‑Diketonate Complex: Structure and Spectroscopic Characterization of [Am(acac)2(μ-acac)(H2O)]2
Source: Inorg Chem. 2026 Aug 23;65(36):20733–9. doi: 10.1021/acs.inorgchem.6c01922 (PMC13580188; doi:10.1021/acs.inorgchem.6c01922)
Supplement: Supplementary file 1 [file ic6c01922_si_001.pdf]

SUPPORTING INFORMATION

Solid−State Luminescence of an Americium(III) β−diketonate Complex: Structure and Spectroscopic Characterization of $[Am(acac)_2(\mu\text{-}acac)(H_2O)]_2$

Thomas L. McCusker,[a] David Schnable,[b] Ana Arteaga,[b] Jeffery A. Bertke,[a] Robert G. Surbella III,[b,] Karah E. Knope [a,]**

[a] Department of Chemistry, Georgetown University, 37th and O Streets NW, Washington, D.C. 20057, United States

[b] Pacific Northwest National Laboratory, 902 Battelle Boulevard, Richland, W.A. 99354, United States

*Correspondence to karah.knope@georgetown.edu or robert.surbella@pnnl.gov

Table of Contents

1. Photographs of Am_2 2

2. Crystallographic Refinement Details 3

3. Thermal Ellipsoid Plot of Am_2 5

4. Powder X−Ray Diffraction of Am_2 (pXRD) 6

5. Full Vibrational Spectrum of Am_2 8

6. Additional Optical Spectra 10

7. References 17

1. Photographs of Am_2

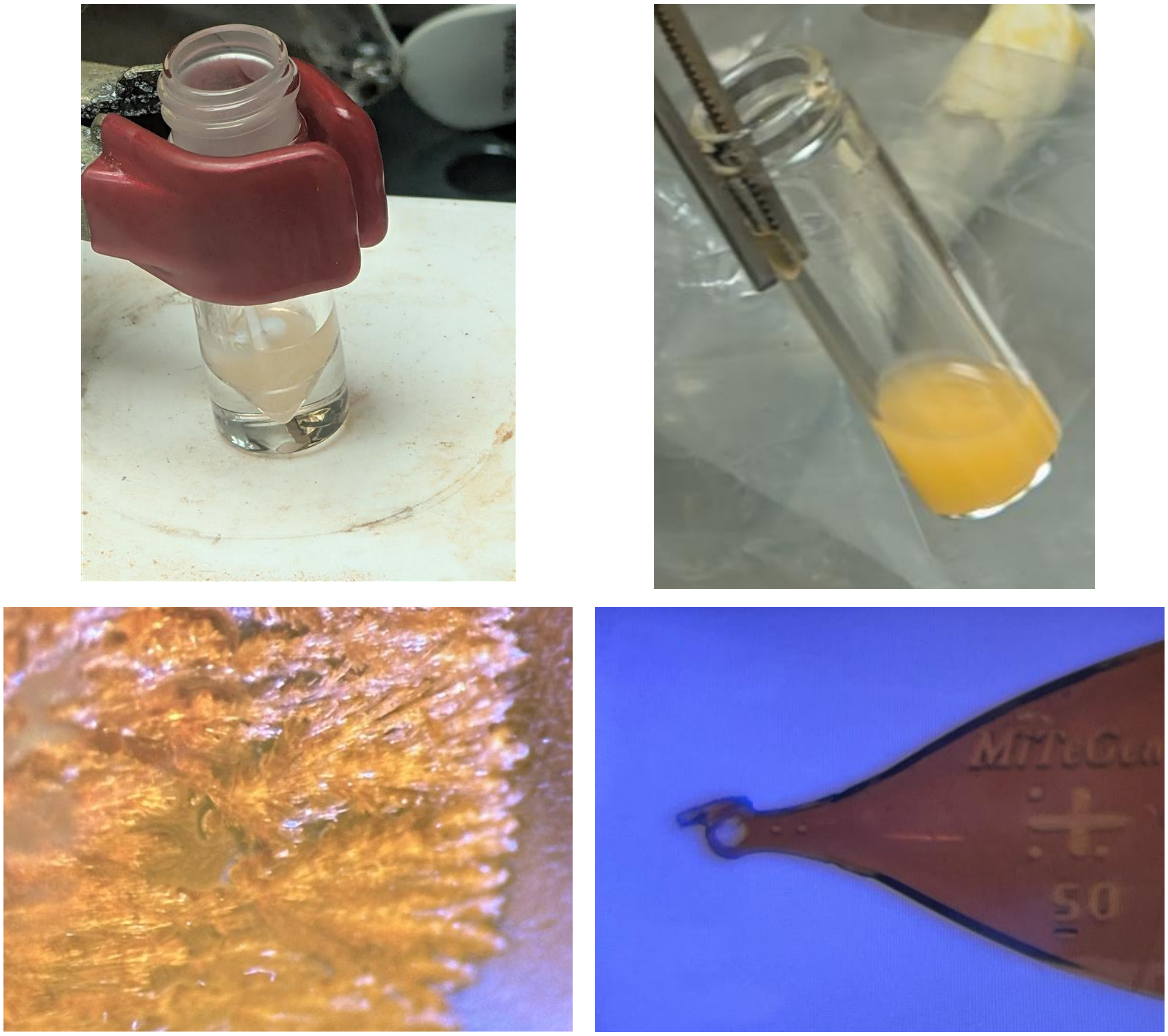

Figure S1. Photographs of the Am(III) stock solution (top left), the $\mathbf{Am_2}$ reaction solution (top right), $\mathbf{Am_2}$ crystals grown on the walls of the glass reaction vial (bottom left) and a single crystal mounted on a MiTeGen micromount (bottom right).

2. Crystallographic Refinement Details

A structural model consisting of one half of the target dimer per asymmetric unit was developed. The water H atoms were located in the difference map, and the O-H distances were restrained using a DFIX command to be 0.88 (esd 0.01) Å. Methyl H atom positions, R-CH_3, were optimized by rotation about R-C bonds and refined with idealized C-H, R--H and H--H distances using the AFIX 137 command. Remaining H atoms were included as riding idealized contributors. Methyl and water H atom U's were assigned as 1.5 times the isotropic displacement parameter, U_{eq}, of the carrier atom; remaining H atom U's were assigned as 1.2 times carrier U_{eq}. The data were processed as a four component non−merohedral twin; however, only reflections from the primary domain were used for structure solution and refinement.

Table S1. Crystallographic refinement details for **Am_2**

**Structure**	**Am_2**
**Formula**	$Am_2O_{14}C_{30}H_{46}$
**MW (g/mol)**	1116.67
**Temperature (K)**	100(2)
**Crystal System**	triclinic
**Space Group**	P-1
**λ (Å)**	0.71073
**a (Å)**	7.9743(12)
**b (Å)**	9.9475(15)
**c (Å)**	12.778(2)
**α (º)**	99.639(5)
**β (º)**	105.790(5)
**γ (º)**	107.768(4)
**V (Å^3)**	893.5(2)
**Z**	1
**ρ (g/cm^3)**	2.075
**μ (mm^{-1})**	4.323
**R_1**	0.0418
**wR_2**	0.0990
**GOF**	1.131

3. Thermal Ellipsoid Plot of Am2

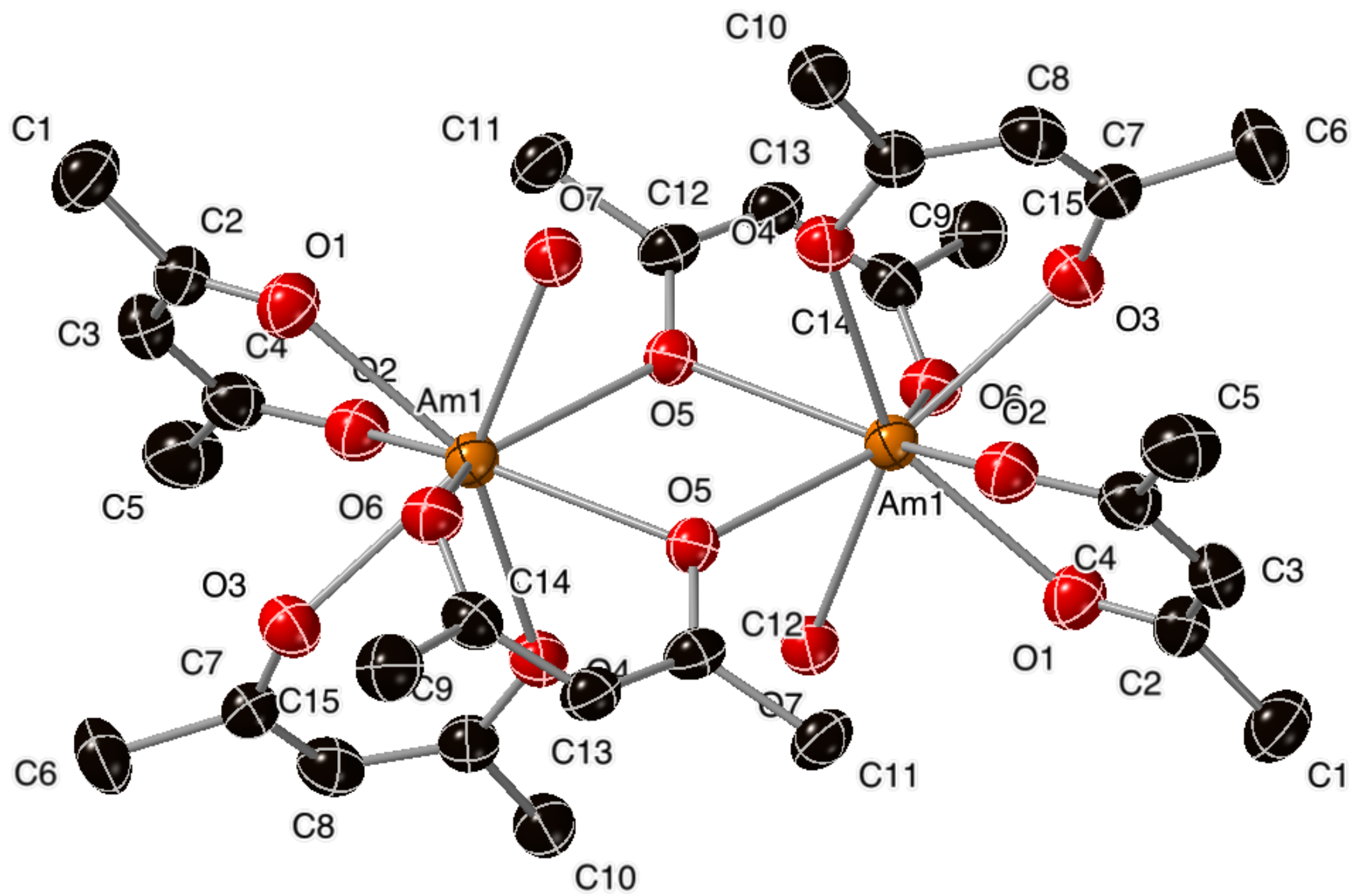

Figure S2: Thermal ellipsoid plot shown at 50% probability level of **Am2**. Americium, oxygen, and carbon are depicted in orange, red, and black respectively. Hydrogen atoms have been omitted for clarity.

4. Powder X−Ray Diffraction of **Am_2** (pXRD)

Powder X−ray diffraction data for the bulk reaction product that yielded **Am_2** were collected and compared to the pattern calculated from the CIF (**Figure S3**). Good agreement between the two patterns indicates that the single crystal used for structure determination is representative of the bulk material. Several additional peaks in the powder pattern (2θ = 12°, 21°, 24°, and 25°) are consistent with triethylamine hydrochloride (TEA•HCl).[1] The salt co−crystallized with **Am_2** and attempts to wash away TEA•HCl led to dissolution of the target compound. Differences in the intensity of the calculated and observed peaks can be attributed to sample preparation method as grinding of the sample to avoid preferred orientation was not possible due to radiological considerations. As such, all spectroscopic characterization of **Am_2** contained TEA•HCl.

Additionally, a low intensity, but sharp peak is present at 7° 2θ that is not accounted for by the simulated pattern. This peak is likely attributed to a minor, unidentified phase that either grows in over the course of the data collection as the powder pattern shown in **Figure S3** is the average of 10 scans collected over approximately 6 hours, or a solvate of **Am_2**.

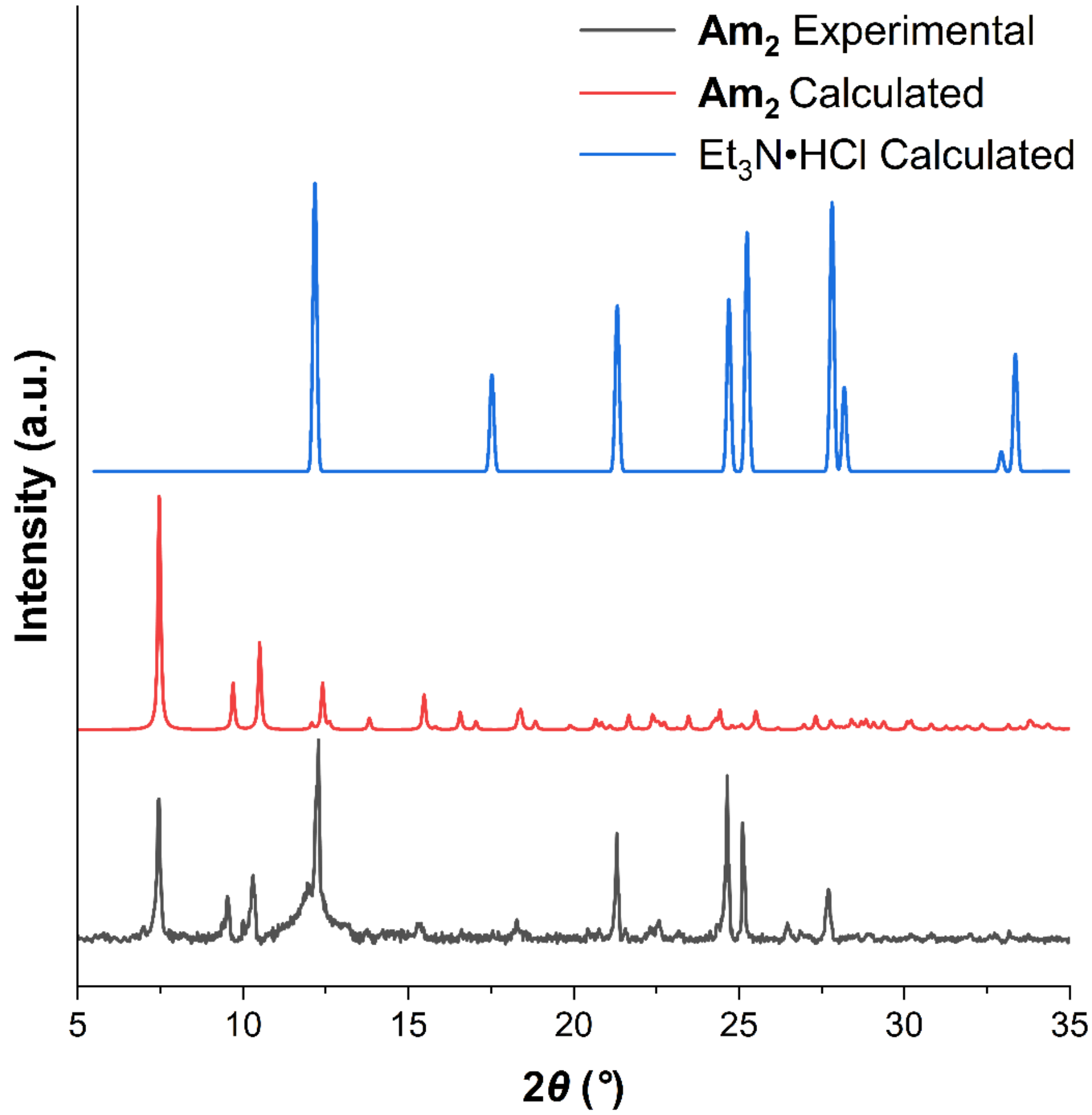

Figure S3: The powder X−ray diffraction pattern of the reaction product (black) compared to the calculated patterns for TEA•HCl (blue) and **Am**$_2$ (red).

5. Full Vibrational Spectrum of **Am2**

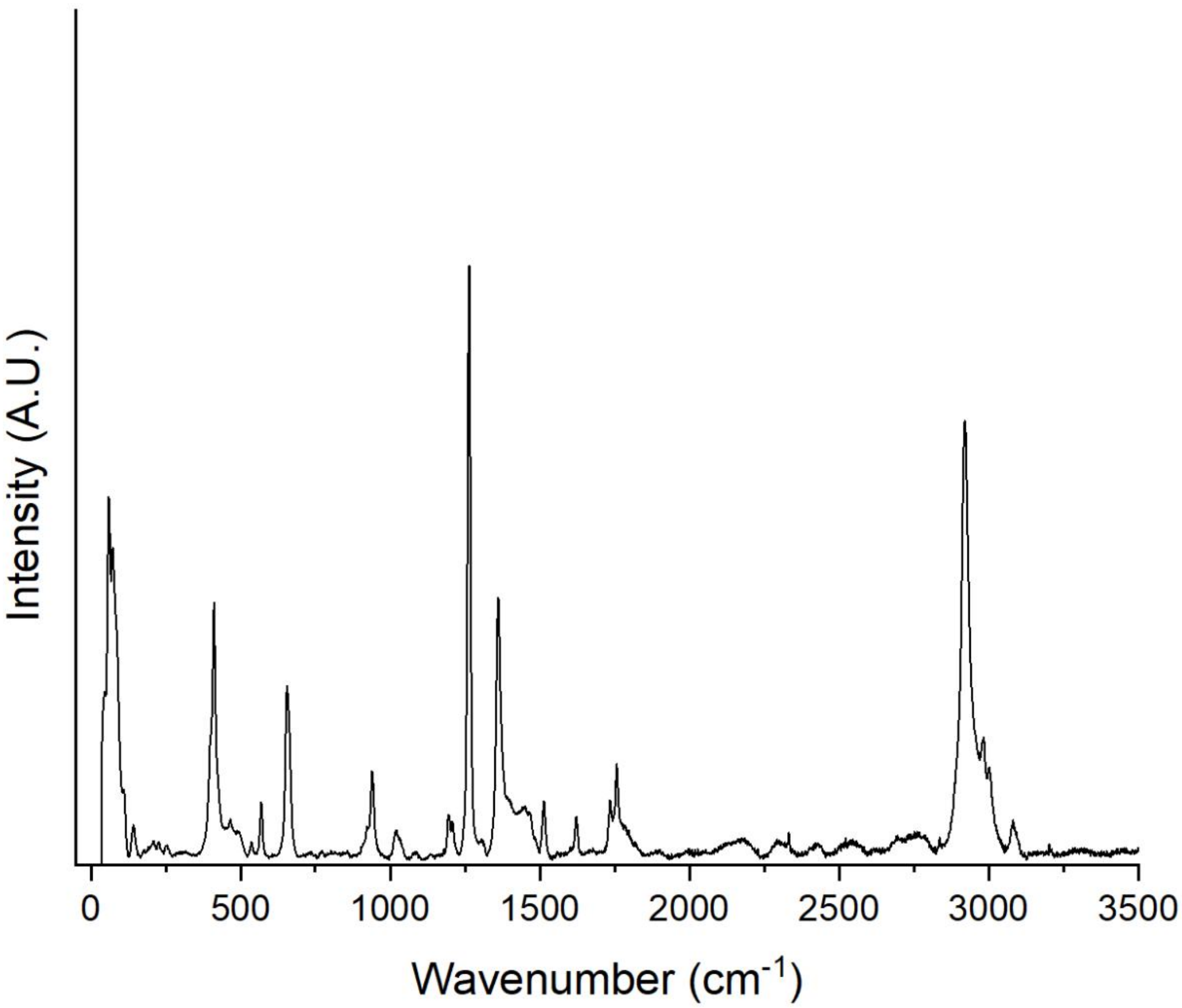

Figure S4: Raman spectrum of **Am2** collected over 50−3500 cm^{-1}.

Peak Analysis

Data Set: **Am_2** Raman spectrum 120–2000 cm^{-1}
BaseLine:Constant

Chi^2=2.97986E+04 Adj. R-Square=9.95559E-01 # of Data Points=1083

SS=2.86960E+07 Degrees of Freedom=963

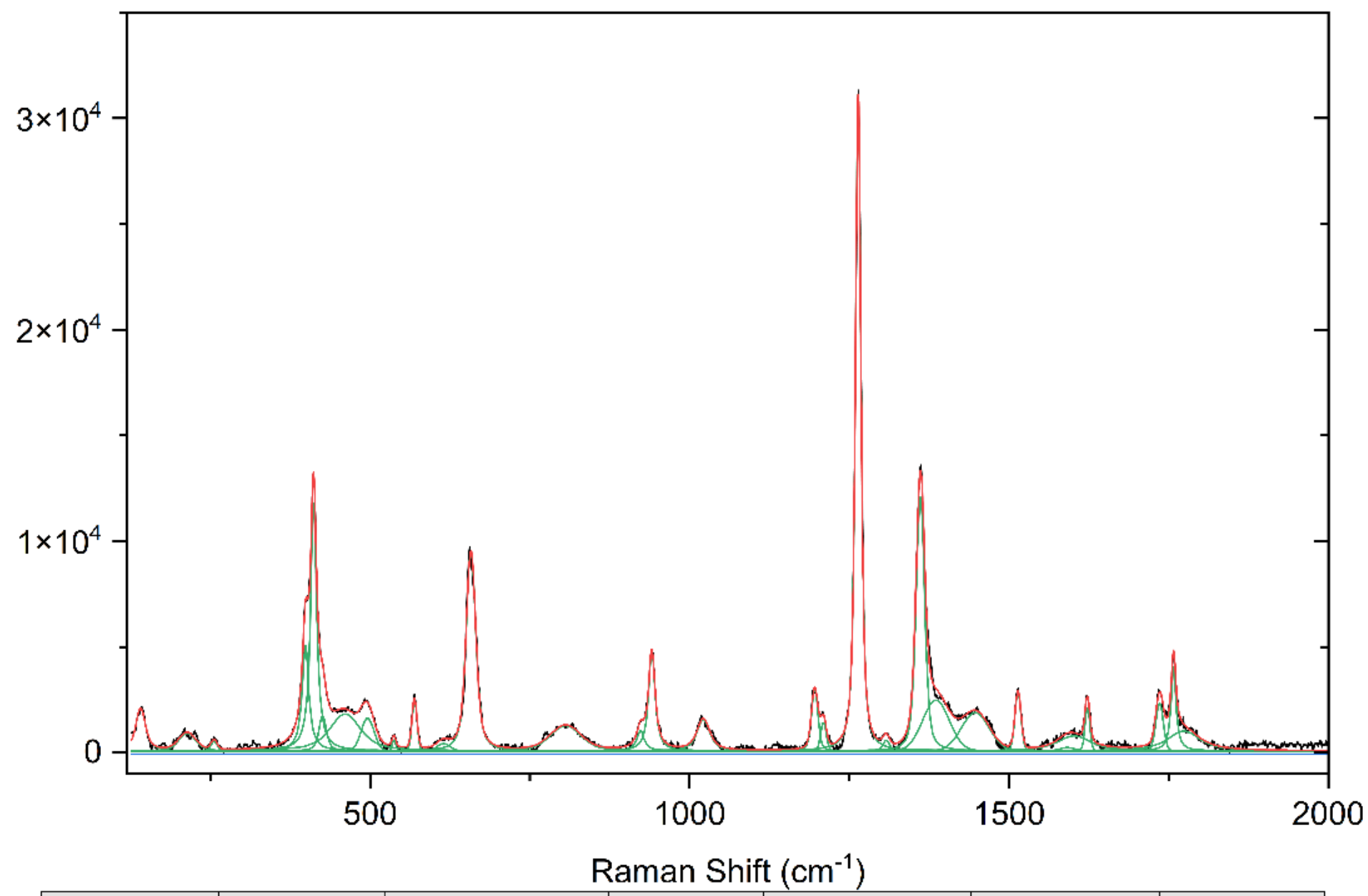

Peak Index	Peak Type	Area Intg	FWHM	Max Height	Center Grvty	Area IntgP
1	Voigt	43485.56451	18.11746	1989.83835	140.59158	1.86417
2	Voigt	31510.8385	31.25347	837.15651	213.42408	1.35083
3	Voigt	5696.85977	11.43595	452.24899	255.56687	0.24422
4	Voigt	94395.67341	12.21325	4974.75934	398.67664	4.04661
5	Voigt	200838.10508	11.0424	11697.50612	411.38619	8.60965
6	Voigt	30744.12871	14.16331	1640.15292	425.58559	1.31796
7	Voigt	138228.33851	57.33832	1751.83863	460.76602	5.92566
8	Voigt	39608.42191	23.69655	1544.37728	495.79242	1.69796
9	Voigt	3897.75315	6.71746	513.3991	537.00552	0.16709
10	Voigt	24003.40374	9.18782	2349.28249	569.23657	1.02899
11	Voigt	8863.01773	23.71668	345.25758	614.82381	0.37995
12	Voigt	217742.39394	17.95314	9410.82588	657.69776	9.33432
13	Voigt	84056.89058	57.36238	1191.74722	805.99272	3.6034
14	Voigt	20841.36965	14.14882	944.06067	923.16949	0.89344
15	Voigt	85701.3092	12.47651	4627.5176	940.83925	3.6739
16	Voigt	49950.0155	23.88881	1488.54747	1021.53932	2.14129
17	Voigt	42560.85995	12.20791	2887.37745	1195.88681	1.82453
18	Voigt	14864.10562	10.43763	1337.84149	1209.47977	0.6372
19	Voigt	430297.27698	10.22304	31068.85748	1263.88594	18.44625
20	Voigt	6652.59256	12.26891	492.97862	1307.67972	0.28519
21	Voigt	239759.10432	16.08858	11996.1054	1361.19262	10.27814
22	Voigt	126108.47308	48.94882	2401.20634	1385.64503	5.4061
23	Voigt	98229.77351	50.53108	1812.3196	1447.12983	4.21097
24	Voigt	33857.27372	11.20827	2737.9856	1514.01897	1.45141
25	Voigt	4952.64906	27.44967	160.29145	1590.94433	0.21231
26	Voigt	63509.95894	62.49254	713.08049	1601.94196	2.72258
27	Voigt	20521.57281	9.08526	2030.2869	1622.65908	0.87973
28	Voigt	33668.5719	13.68086	2245.86528	1735.58934	1.44333
29	Voigt	57476.61368	9.27499	3989.35738	1757.47016	2.46394
30	Voigt	80685.91041	60.87568	936.67775	1773.57925	3.45889

Figure S5: Peak deconvolution of the Raman spectrum of **Am_2** in the 120–2000 cm^{-1} region.

6. Additional Optical Spectra

Peak Analysis

Data Set: **Am_2** UV region absorption bands

BaseLine:Constant

Chi^2=1.58359E-05 Adj. R-Square=9.98802E-01 # of Data Points=191

SS=2.39122E-03 Degrees of Freedom=151

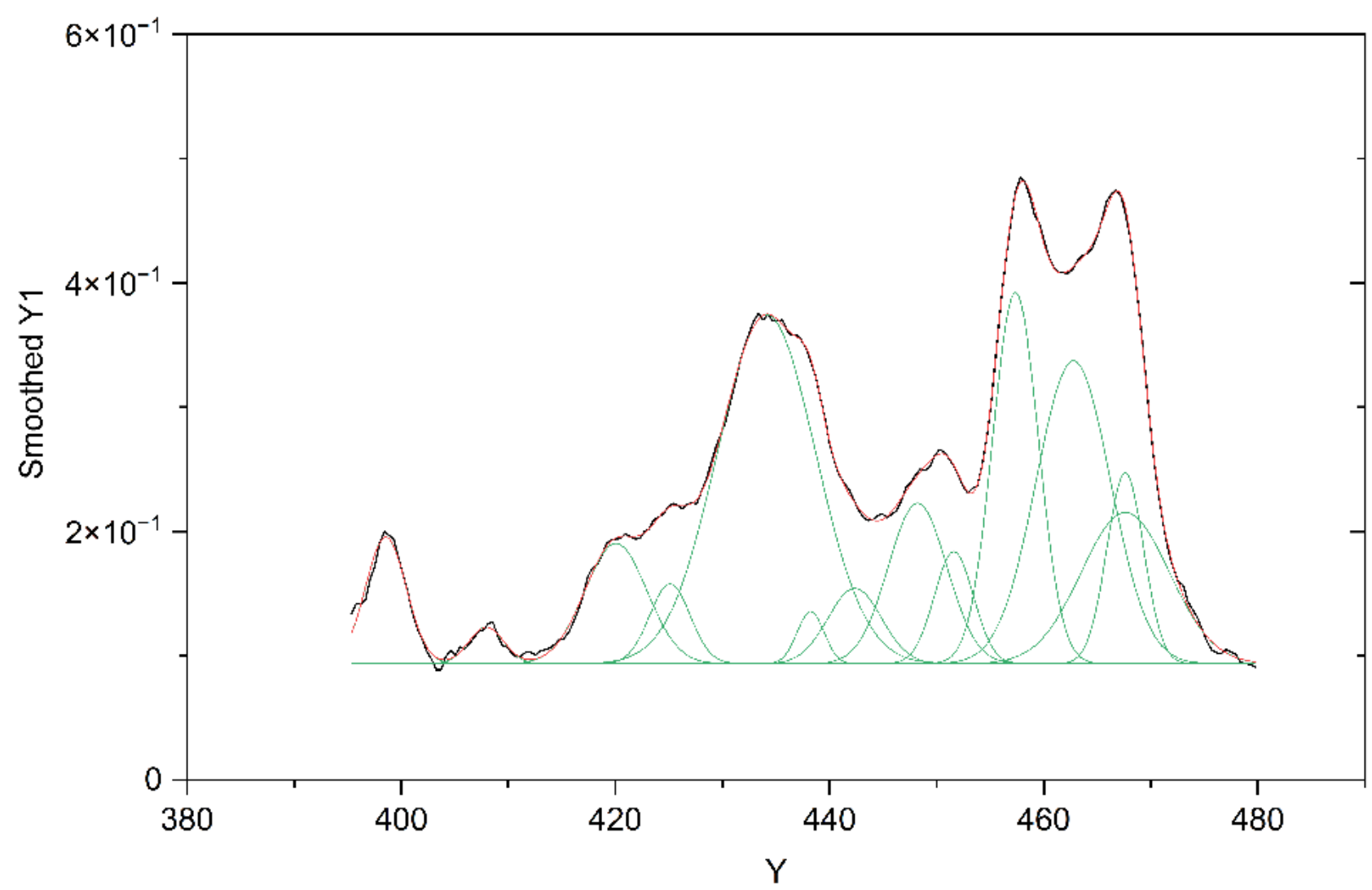

Fitting Results

Peak Index	Peak Type	Area Intg	FWHM	Max Height	Center Grvty	Area IntgP
1	Gaussian	0.46339	4.47316	0.1019	398.55181	3.77971
2	Gaussian	0.70397	6.83564	0.09675	420.03863	5.74212
3	Gaussian	0.30558	4.46546	0.06429	425.08073	2.49251
4	Gaussian	3.22525	10.79278	0.28074	434.10405	26.30747
5	Gaussian	0.13219	2.95203	0.04207	438.21695	1.07821
6	Gaussian	0.38159	5.91026	0.06065	442.34497	3.11254
7	Gaussian	0.41262	4.30306	0.09008	451.60131	3.36566
8	Gaussian	1.60172	5.04013	0.29855	457.35389	13.06478
9	Gaussian	0.66015	4.03996	0.15351	467.60444	5.3847
10	Gaussian	0.12046	3.84527	0.02943	407.93933	0.98252
11	Gaussian	0.88626	6.44829	0.12912	448.2076	7.229
12	Gaussian	1.25023	9.6645	0.12172	467.60444	10.19779
13	Gaussian	2.11641	8.15984	0.24366	462.76006	17.26298

Figure S6: Gaussian peak deconvolution for the various UV−region absorption bands of **Am_2**.

Peak Analysis

Data Set: **Am_2** 504 nm absorption band

BaseLine:Straight

Chi^2=2.86006E-05 Adj. R-Square=9.99687E-01 # of Data Points=111

SS=2.71705E-03 Degrees of Freedom=95

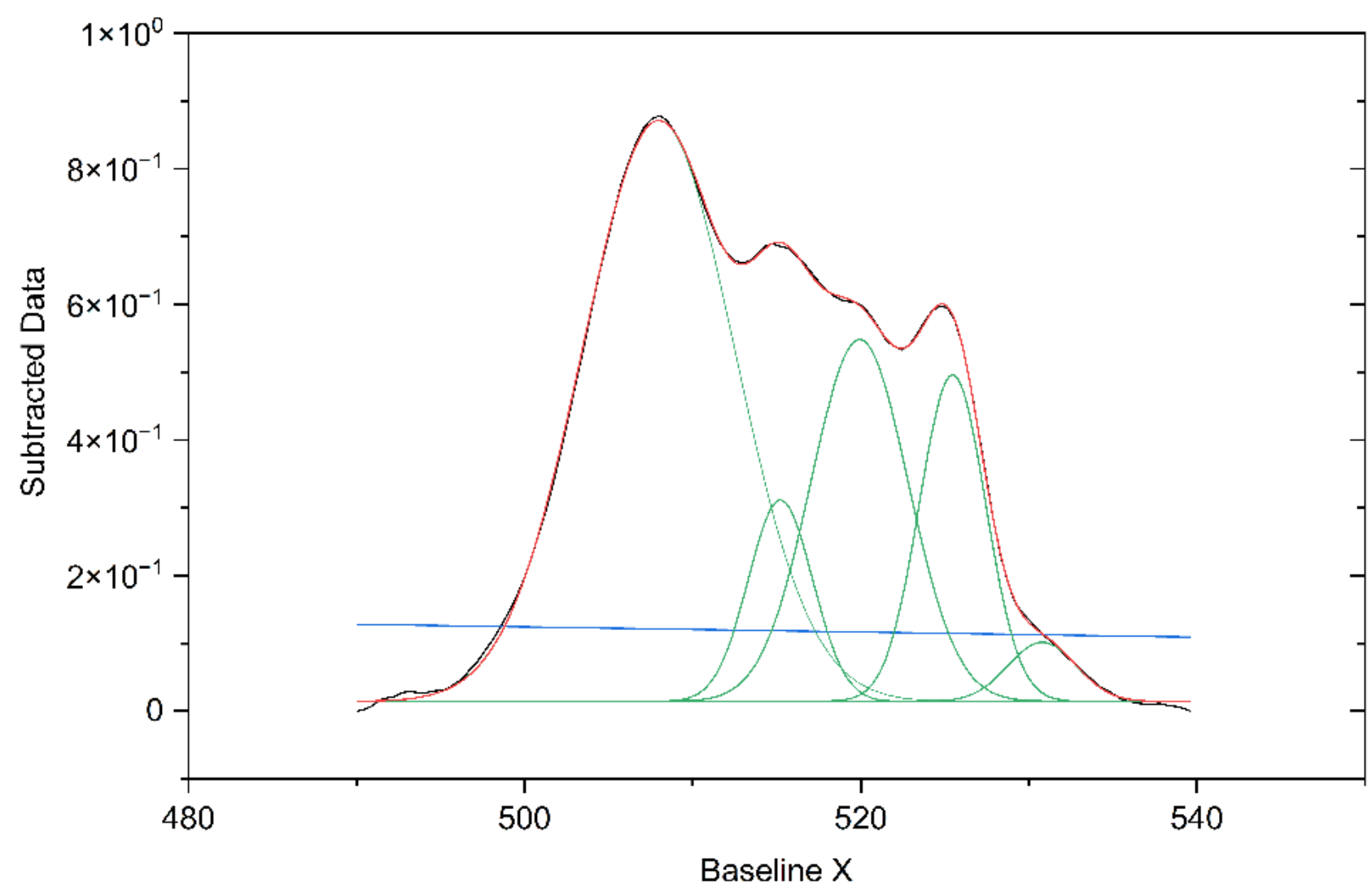

Fitting Results

Peak Index	Peak Type	Area Intg	FWHM	Max Height	Center Grvty	Area IntgP
1	Gaussian	9.72918	10.66874	0.85674	507.96331	54.49761
2	Gaussian	1.42583	4.51038	0.29698	515.21197	7.98671
3	Gaussian	2.37996	4.64294	0.48155	525.45526	13.33124
4	Gaussian	3.87412	6.81613	0.53395	519.92171	21.70076
5	Gaussian	0.4434	4.77176	0.08729	530.78671	2.48368

Figure S7: Gaussian peak deconvolution for the $^5L_6' \leftarrow {}^7F_0'$ absorption band of **Am_2**.

Peak Analysis

Data Set: **Am_2** 800 nm absorption band

BaseLine:Straight

Chi^2=5.22937E-06 Adj. R-Square=9.99912E-01 # of Data Points=189

SS=8.57617E-04 Degrees of Freedom=164

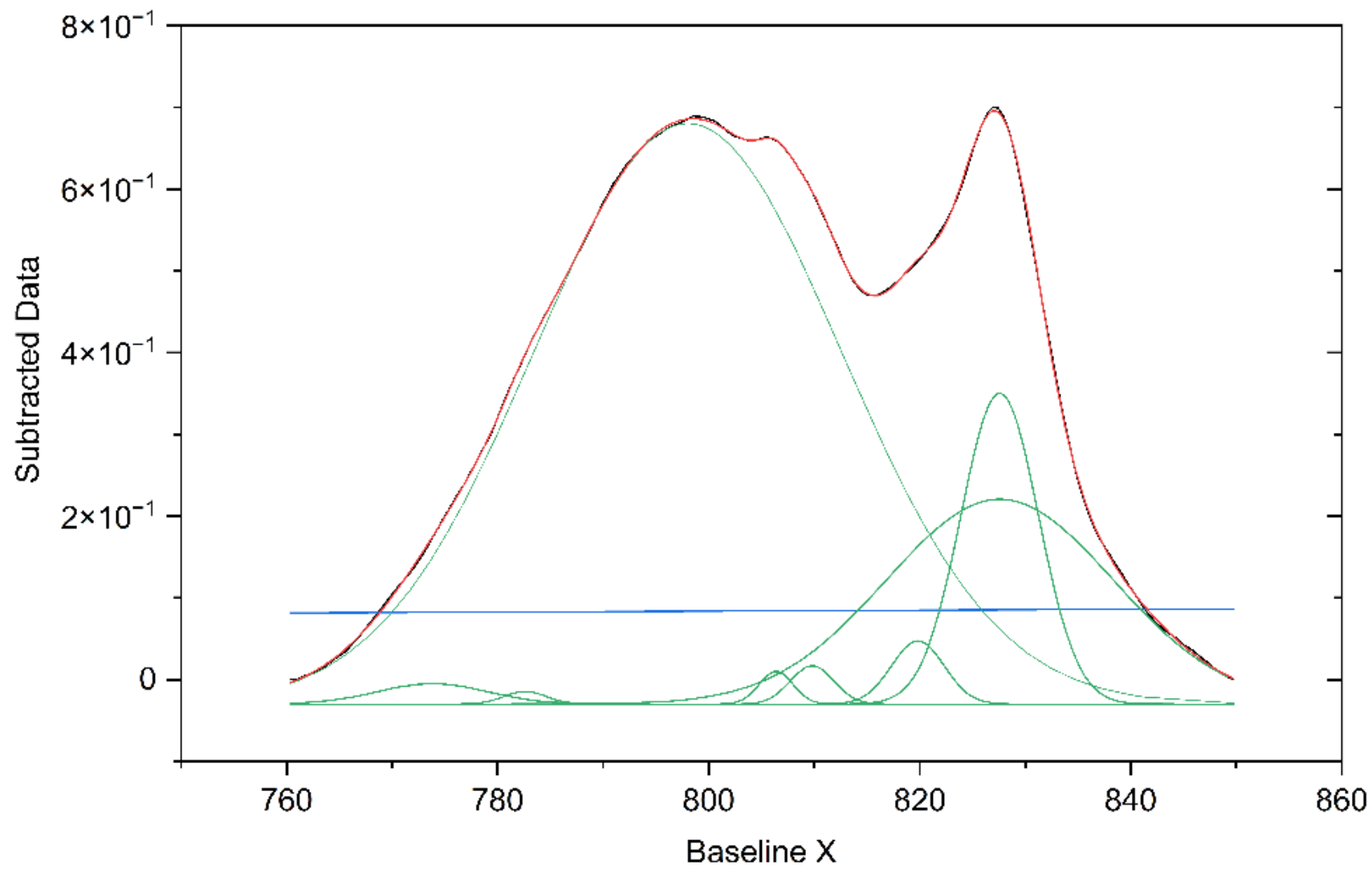

Fitting Results

Peak Index	Peak Type	Area Intg	FWHM	Max Height	Center Grvty	Area IntgP
1	Gaussian	25.84846	34.36709	0.71019	798.00196	69.35509
2	Gaussian	3.50733	8.66601	0.38021	827.5638	9.41067
3	Gaussian	6.61871	25.30594	0.25058	827.57734	17.75895
4	Gaussian	0.4856	5.90294	0.07728	819.81435	1.30294
5	Gaussian	0.16085	3.77669	0.04001	806.37799	0.43158
6	Gaussian	0.24204	4.84651	0.04692	809.79358	0.64942
7	Gaussian	0.32752	12.34192	0.02506	773.80261	0.87878
8	Gaussian	0.07922	4.77163	0.0156	782.59836	0.21256

Figure S8: Gaussian peak deconvolution for the $^{7}F_{6}'\leftarrow{}^{7}F_{0}'$ absorption band of **Am_2**.

Peak Analysis

Data Set: **Am_2** NIR absorption bands

BaseLine:Constant

Chi^2=4.13716E-05 | Adj. R-Square=9.95055E-01 | # of Data Points=255

SS=9.76369E-03 | Degrees of Freedom=236

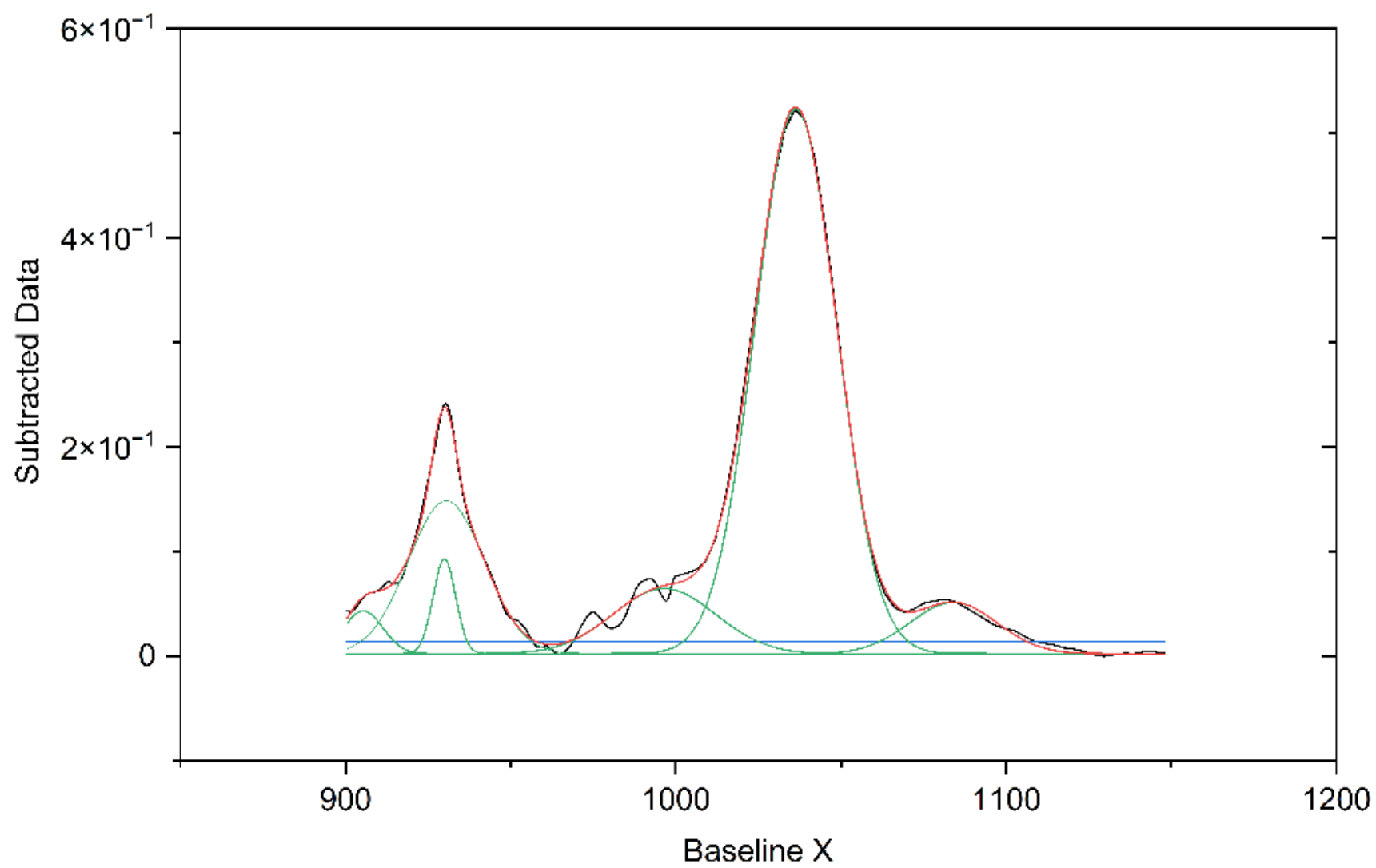

Fitting Results

Peak Index	Peak Type	Area Intg	FWHM	Max Height	Center Grvty	Area IntgP
1	Gaussian	4.1935	27.08685	0.14611	930.45929	16.38801
2	Gaussian	16.10599	29.0621	0.52063	1036.3123	62.94142
3	Gaussian	0.77448	8.03971	0.0905	929.91356	3.02664
4	Gaussian	1.61637	30.96589	0.04904	1084.01961	6.3167
5	Gaussian	0.4821	13.95548	0.0409	905.32918	1.88401
6	Gaussian	2.41641	36.5026	0.06219	996.45521	9.44322

Figure S9: Gaussian peak deconvolution for the $^7F_{4-5}'\leftarrow{}^7F_0'$ absorption bands of **Am_2**.

Peak Analysis

Data Set: **Am_2** 690 emission band

BaseLine:End Points Weighted

Chi^2=4.90546E+02 Adj. R-Square=9.99915E-01 # of Data Points=81

SS=3.09044E+04 Degrees of Freedom=63

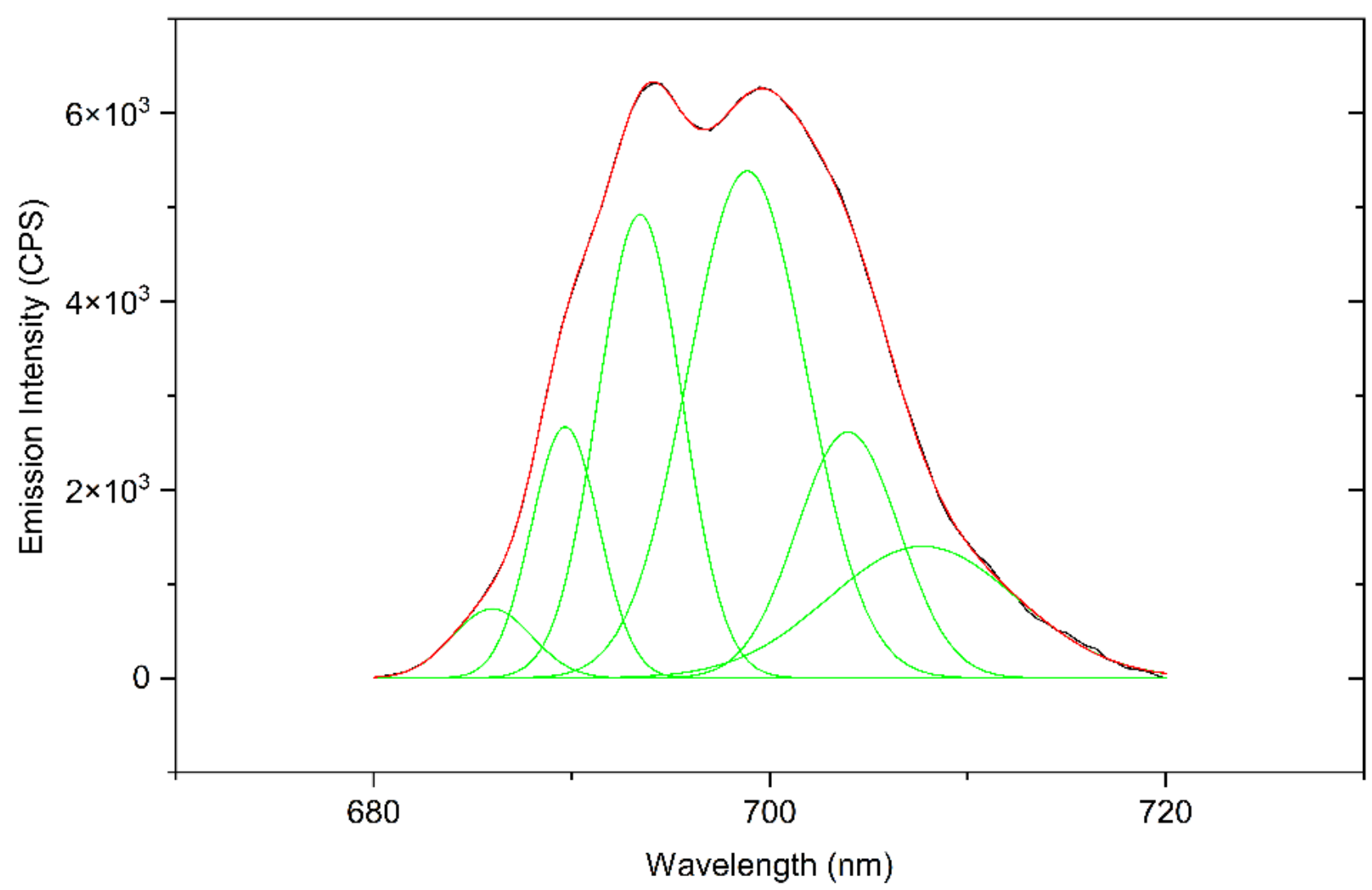

Fitting Results

Peak Index	Peak Type	Area Intg	FWHM	Max Height	Center Grvty	Area IntgP
1	Gaussian	11447.55612	4.03072	2668.07613	689.67352	9.92261
2	Gaussian	26208.4782	5	4924.24429	693.45466	22.71721
3	Gaussian	40396.26892	7.04532	5386.5223	698.86582	35.01503
4	Gaussian	16658.02032	11.22793	1400.8355	707.73136	14.43898
5	Gaussian	3692.75265	4.72782	734.82172	685.98751	3.20084
6	Gaussian	16965.28821	6.09685	2614.10953	703.95483	14.70532

Figure S10: Gaussian peak deconvolution of the $^5D_1' \rightarrow {}^7F_1'$ emission band of **Am_2**.

Peak Analysis

Data Set: **Am_2** 830 emission band

BaseLine:End Points Weighted

Chi^2=1.20904E+04 Adj. R-Square=9.99778E-01 # of Data Points=86

SS=8.82600E+05 Degrees of Freedom=73

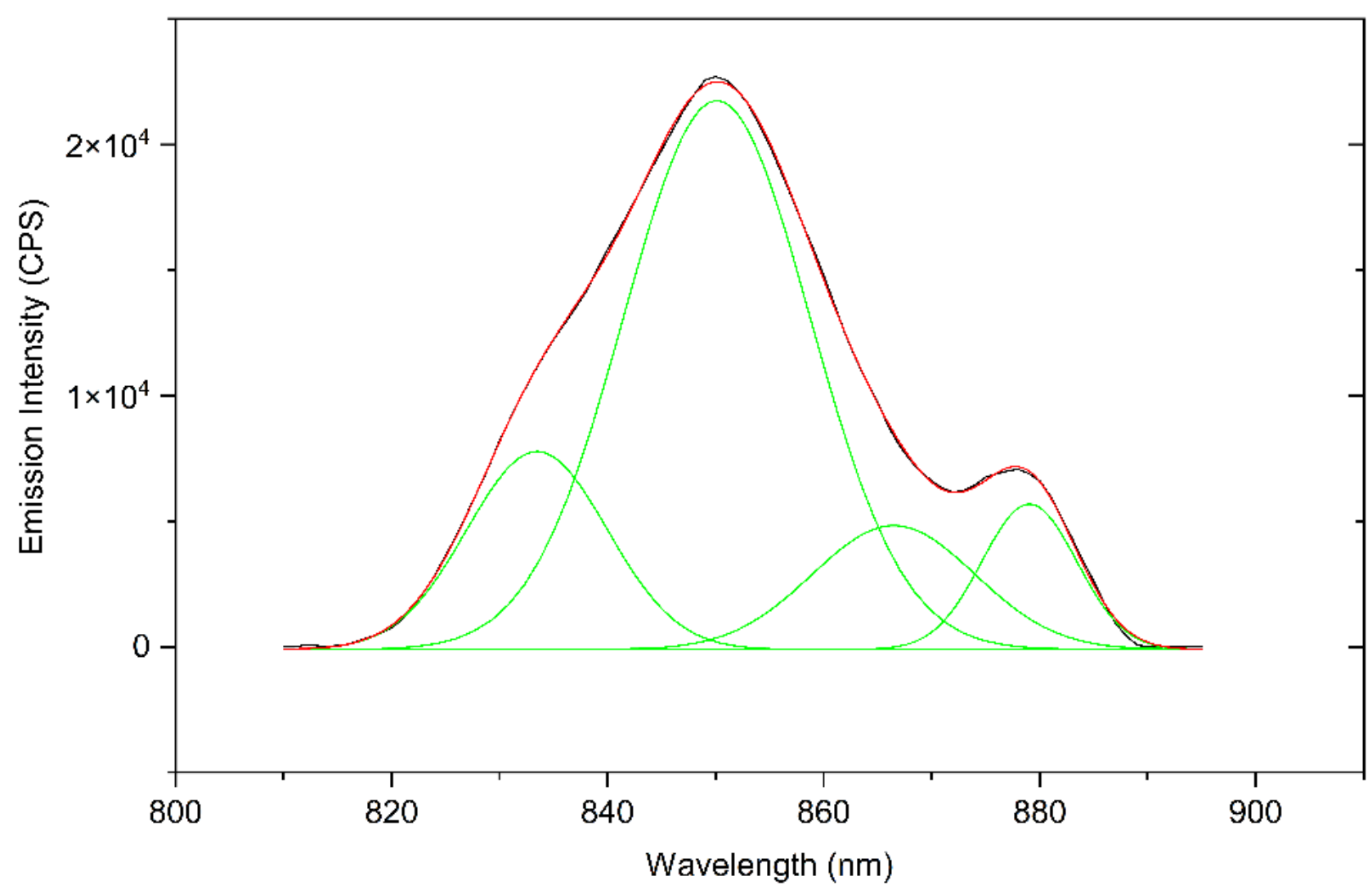

Fitting Results

Peak Index	Peak Type	Area Intg	FWHM	Max Height	Center Grvty	Area IntgP
1	Gaussian	128413.46489	15.30597	7882.87035	833.4627	16.93717
2	Gaussian	472591.64762	20.30991	21859.82046	850.10574	62.33277
3	Gaussian	93344.7183	17.75941	4938.13533	866.47769	12.31176
4	Gaussian	63825.47442	10.35327	5792.24168	879.03787	8.4183

Figure S11: Gaussian peak deconvolution of the $^5D_1'\rightarrow{}^7F_2'$ emission band of **Am_2**.

Peak Analysis

Data Set: **Am_2** 1030 emission band

BaseLine:End Points Weighted

Chi^2=4.53317E-14 Adj. R-Square=9.99861E-01 # of Data Points=71

SS=2.62924E-12 Degrees of Freedom=58

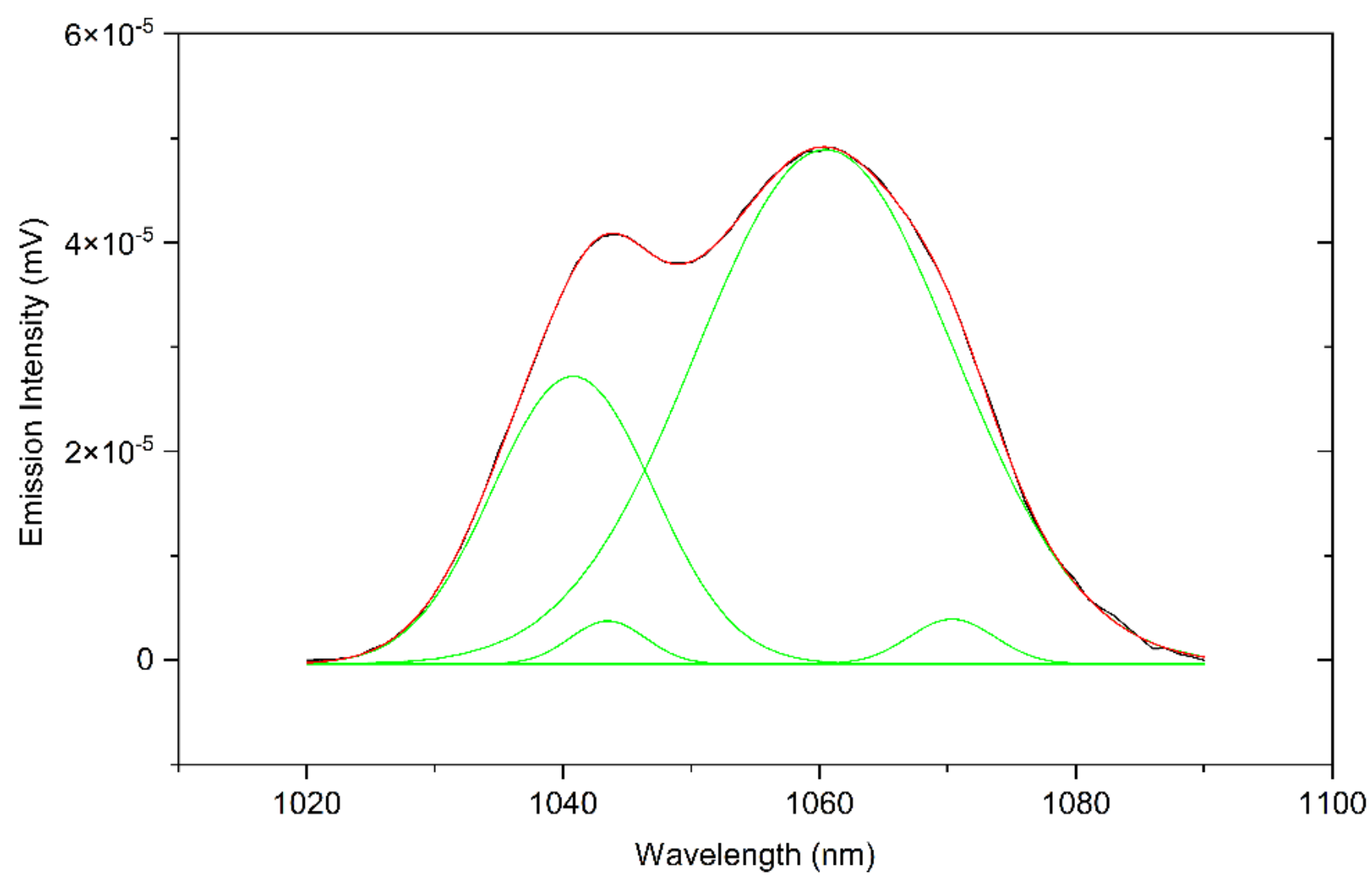

Fitting Results

Peak Index	Peak Type	Area Intg	FWHM	Max Height	Center Grvty	Area IntgP
1	Gaussian	4.3396E-4	14.80237	2.75545E-5	1040.78442	24.87162
2	Gaussian	3.05514E-5	6.95506	4.12665E-6	1043.48461	1.751
3	Gaussian	0.00124	23.77415	4.92523E-5	1060.46618	71.31105
4	Gaussian	3.60534E-5	7.88366	4.29622E-6	1070.3391	2.06634

Figure S12: Gaussian peak deconvolution of the $^5D_1' \rightarrow {}^7F_3'$ emission band of **Am_2**.

7. References

(1) Churakov, A. V; Howard, J. A. K. Redetermination of Triethylammonium Chloride in the Space Group P31c. *Crystal Structure Communications* **2004**, *60* (8), o557–o558.
